# Supplementary material for: Starch and Sucrose Metabolism and Plant Hormone Signaling Pathways Play Crucial Roles in Aquilegia Salt Stress Adaption
Source: Int J Mol Sci. 2023 Feb 16;24(4):3948. doi: 10.3390/ijms24043948 (PMC9966690; doi:10.3390/ijms24043948)
Supplement: Supplementary file 1 [file ijms-24-03948-s001.zip › Figure S2.pdf]

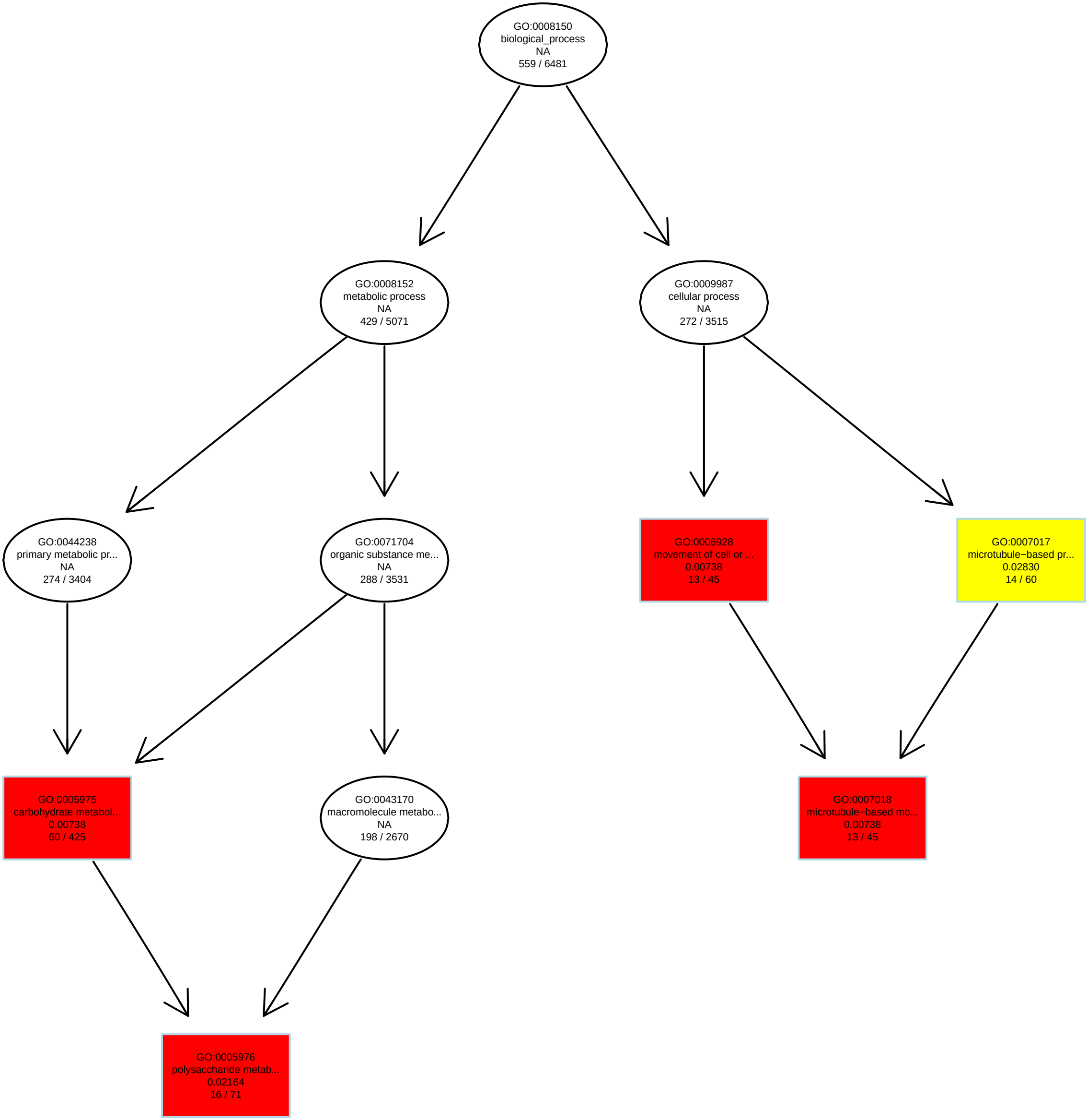

Figure S2a. Biological process in GO enrichment of *Aquilegia vulgaris* at 24 h

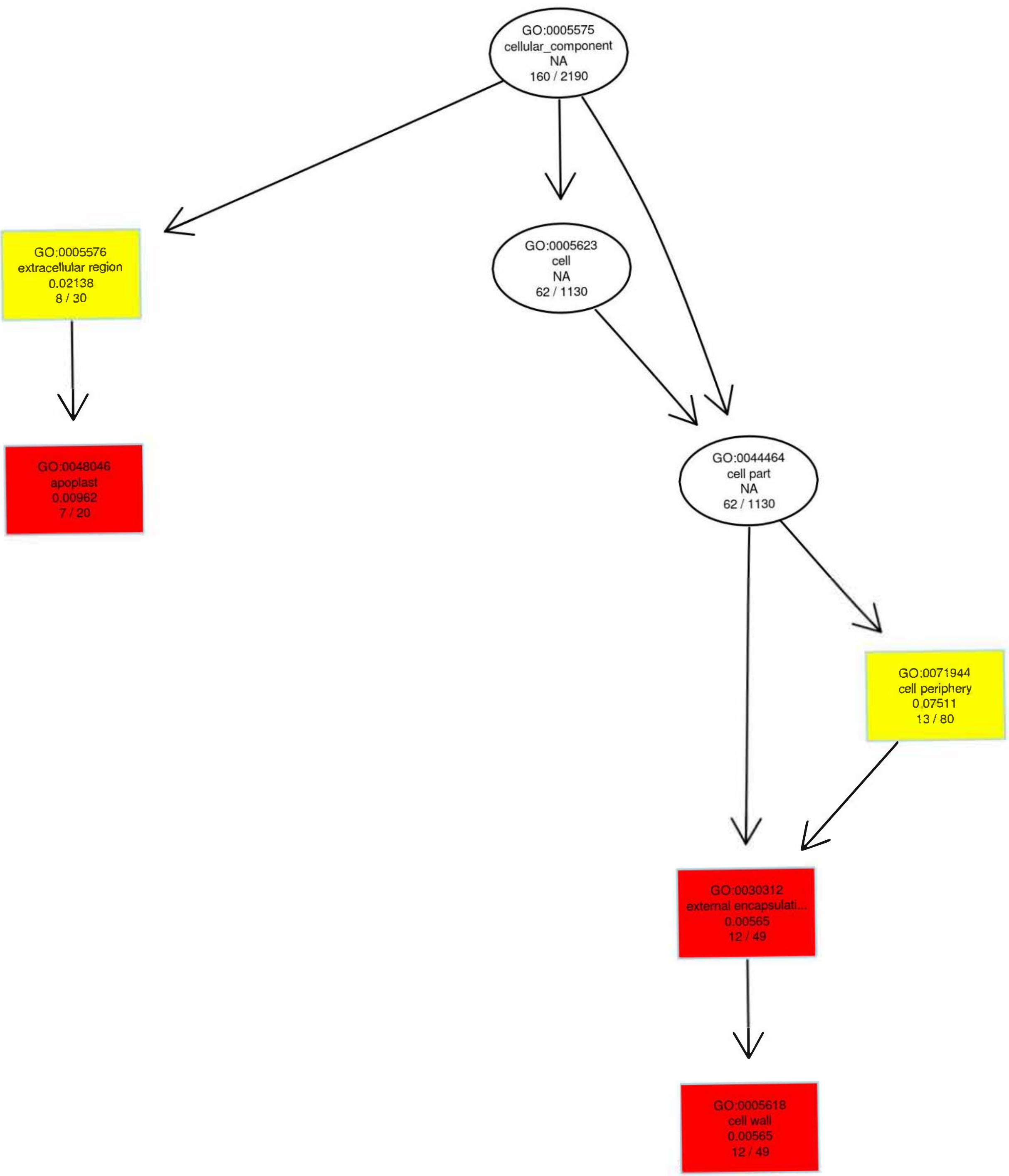

Figure S2b. Cellular component in GO enrichment of *Aquilegia vulgaris* at 24 h

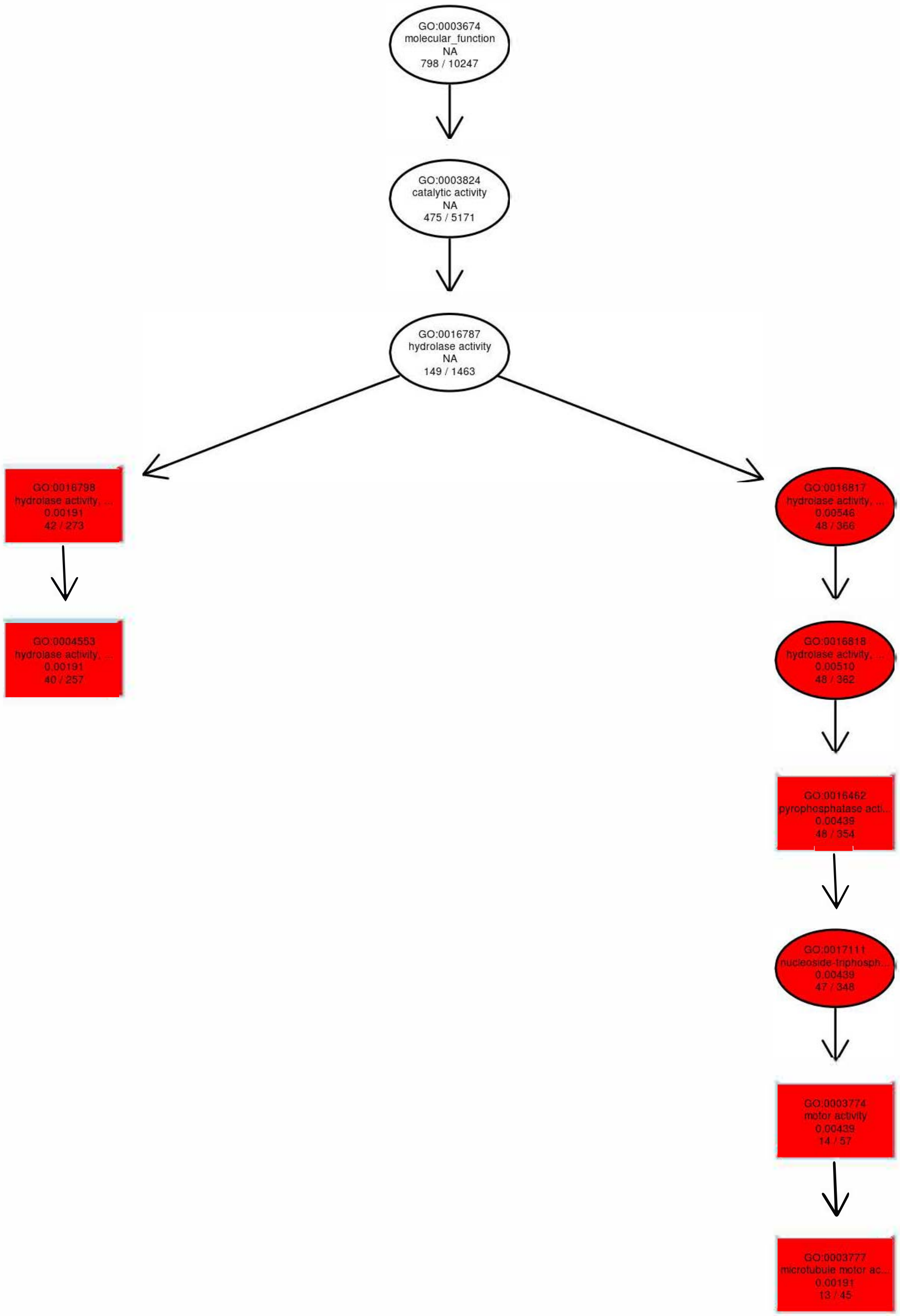

Figure S2c. Molecular function in GO enrichment of *Aquilegia vulgaris* at 24 h
